# Neurogenic Stunned Myocardium Associated with Acute Spinal Cord Infarction: A Case Report

**DOI:** 10.1155/2012/439528

**Published:** 2012-02-28

**Authors:** Gillian A. Beauchamp, Jason T. McMullan, Jordan B. Bonomo

**Affiliations:** ^1^Department of Emergency Medicine, University of Cincinnati, Cincinnati, OH 45267, USA; ^2^Division of Neurocritical Care, Department of Neurosurgery, University of Cincinnati, 231 Albert Sabin Way, ML 0769, MSB 1654, Cincinnati, OH 45267, USA

## Abstract

*Introduction*. Neurogenic stunned myocardium (NSM) is a reversible cardiomyopathy resulting in transient left ventricular apical ballooning presumed to result from catecholamine surge occurring under physiologic stress. Acute spinal cord ischemia is a rare ischemic vascular lesion. We report a case of neurogenic stunned myocardium occurring in the setting of acute spinal cord infarction. *Methods*. Singe case report was used. *Results*. We present the case of a 63-year-old female with a history of prior lacunar stroke, hypertension, chronic back pain, and hypothyroidism who presented with a brief episode of diffuse abdominal and bilateral lower extremity pain which progressed within minutes to bilateral lower extremity flaccid paralysis. MRI of the spinal cord revealed central signal hyperintensity of T2-weighted imaging from conus to T8 region, concerning for acute spinal cord ischemia. Transthoracic echocardiogram was performed to determine if a cardiac embolic phenomenon may have precipitated this ischemic event and showed left ventricular apical hypokinesis and ballooning concerning for NSM. *Conclusion*. Neurogenic stunned myocardium is a reversible cardiomyopathy which has been described in patients with physiologic stress resulting in ventricular apical ballooning. Our case suggests that it is possible for neurogenic stunned myocardium to occur in the setting of acute spinal cord ischemia.

## 1. Introduction

Neurogenic stunned myocardium (NSM) is a reversible cardiomyopathy resulting in transient left ventricular apical ballooning which has been described to occur in the setting of catecholamine release [1, 2] during situations of physiologic stress such as subarachnoid hemorrhage [3–5], reversible posterior leukoencephalopathy [6], atrial fibrillation [7], hemorrhagic cerebral contusion [8], status epilepticus [9], ischemic cerebrovascular accident [10], limbic encephalitis [11], and severe emotional stress [12]. NSM has also been referred to as “broken heart syndrome” [12], “takotsubo cardiomyopathy,” “apical ballooning syndrome,” “neurogenic stress cardiomyopathy,” [13] and “transient left ventricular dysfunction syndrome.” [14] This condition typically presents with mildly elevated cardiac biomarkers [14] and reversible regional-wall motion abnormalities on echocardiogram [15]. Care for NSM involves treatment of the underlying cause and supportive care. 

Acute spinal cord ischemia is a rare ischemic vascular lesion with high morbidity and mortality [16]. To the best of our knowledge, this is the first case report of the occurrence of neurogenic stunned myocardium in the context of acute spinal cord infarction.

## 2. Case Presentation

A 63-year-old female with a history of prior lacunar stroke, hypertension, chronic back pain, and hypothyroidism presented to a community emergency department one hour after the acute onset of severe bilateral lower extremity pain which progressed, within minutes, to bilateral lower extremity generalized weakness and then flaccid paralysis. In addition, within an hour of symptom onset, she developed dull, diffuse abdominal pain with radiation to the bilateral flanks, lasting one hour with subsequent resolution.

At presentation, she was found to have a blood pressure of 131/88 mmHg, tachycardia with a heart rate of 110 beats per minute, oral temperature of 96.8 degrees, F and SpO_2_ of 97% on room air. The patient presented with 0/5 strength and decreased sensation to light touch and areflexia of the bilateral lower extremities. Noncontrast computed tomography (CT) of the head showed a remote lacunar infarct. CT angiography of the abdomen and pelvis showed no evidence of pulmonary embolus, abdominal aortic aneurysm, or aortic dissection. Surface electrocardiogram showed sinus tachycardia without ischemia.

**Figure 1:**
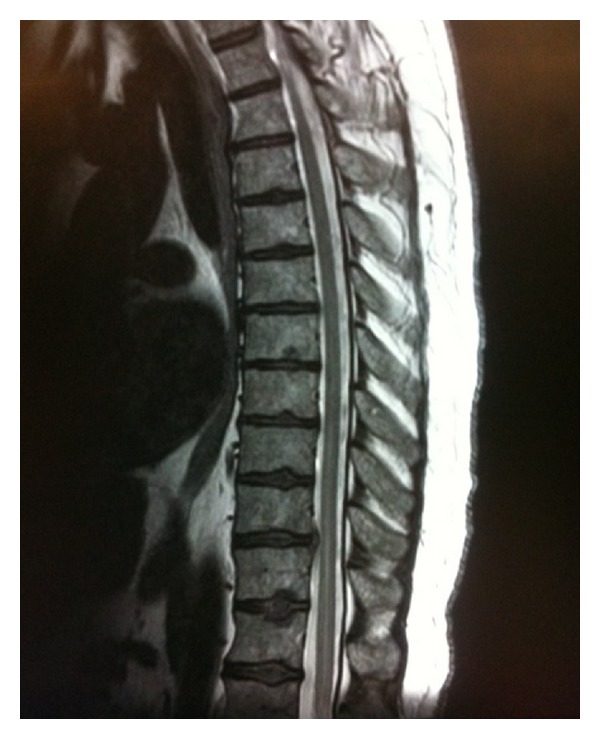
MRI thoracic spine showing acute spinal cord infarction. Sagittal T2-weighted MRI of the patient's thoracic spine demonstrating nonspecific central intrinsic signal hyperintensity of the spinal cord located in conus region with extension to T8 level. Multilevel thoracic disc disease at T1 and T10-T11 is also present.

**Figure 2:**
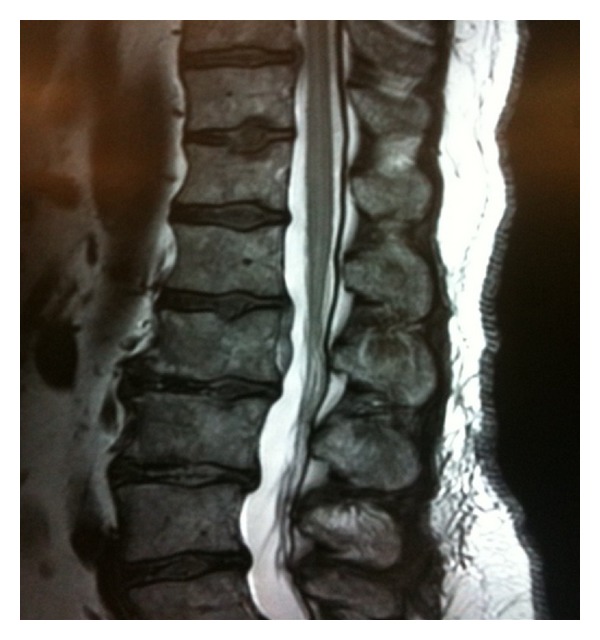
MRI of the lumbar spine showing acute spinal cord infarction. Sagittal T2-weighted MRI of the patient's lumbar spine showing a nonspecific central intrinsic signal hyperintensity of the spinal cord located in conus region with extension to T8 level. Image also shows multilevel degenerative disc disease with noncompressive disc herniation at L1-L2.

**Figure 3:**
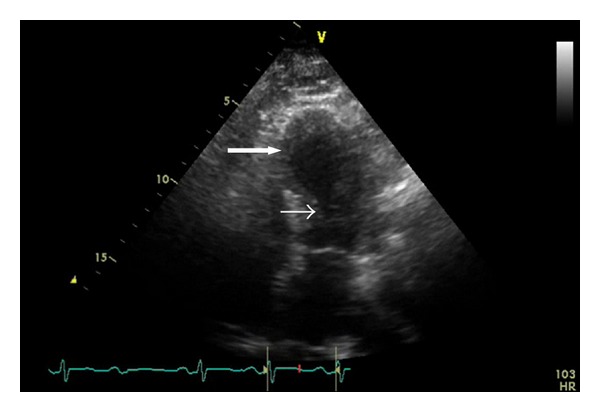
Transthoracic echocardiogram. The patient's apical four-chamber transthoracic echocardiogram at end systole showing left ventricle, left atrium, and mitral valve. There is left ventricular basilar contraction (small arrow), with near apposition of the endomyocardium, and severe apical hypokinesia (large arrow) with apical ballooning.

**Figure 4:**
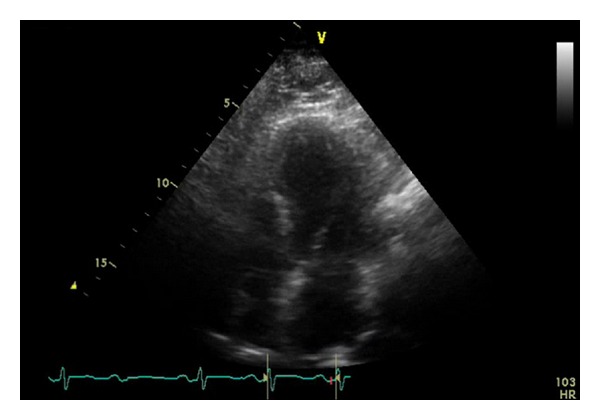
Transthoracic echocardiogram. The patient's transthoracic echocardiogram in diastole showing neurogenic stunned myocardium. Apical four-chamber view showing left ventricle, left atrium, and mitral valve during diastole. Note the uniformity of the endomyocardium resulting in a “bullet shape” of the left ventricle in which all three segments of the ventricle: base, mid, and apex relax equally.

The patient was transferred to a tertiary care center for advanced imaging and care, where a T2-weighted magnetic resonance image (MRI) showed a nonspecific central intrinsic signal hyperintensity in the conus region extending to the level of T8, concerning for acute spinal cord ischemia (Figures 1 and 2). Transthoracic echocardiogram, performed to determine if there was a cardiac source of emboli, showed atypical dyskinesia with left ventricular apical ballooning and an ejection fraction of 35%, a characteristic of neurogenic stunned myocardium (Figures 3 and 4). Cardiology was consulted, and a diagnosis of takotsubo cardiomyopathy was made, with no further recommendation for heparinization or catheterization. Pertinent laboratory results are shown in Table 1.

The patient was admitted to the neurology inpatient service for supportive care including physical and occupational therapy. During admission, the patient was evaluated for stroke risk factors, and statin therapy was subsequently initiated for hyperlipidemia. No other specific underlying etiology of the spinal cord ischemia was found. Cerebrospinal fluid studies were performed to rule out other etiologies for paralysis and were unremarkable. The patient was started on low-dose beta-blocker and ACE-inhibitor therapy per cardiology recommendations, and the patient was discharged to a rehabilitation facility with the recommendation to have a transthoracic echocardiogram six months after discharge. The patient was subsequently lost to followup.

## 3. Discussion

Neurogenic stunned myocardium (NSM) has most recently been explained by the “catecholamine hypothesis,” which describes the underlying activation of sympathetic nervous system activity as the cause of reversible apical ballooning and left ventricular dysfunction [1, 2]. Specifically, catecholamine release from sympathetic nerves innervating the myocardium leads to the development of reversible wall motion abnormalities, typically presenting as apical ballooning on echocardiogram and occurring with mild troponin elevations [3, 5, 17]. Although not performed in this patient, coronary artery disease is ideally ruled out with coronary angiography and resolution of cardiac stun confirmed with a repeated echocardiogram performed two to four weeks after the onset of NSM [2].

The occurrence of NSM in the face of acute spinal cord ischemia suggests underlying physiologic or emotional stress as a possible etiology for this myocardial regional wall motion abnormality. Additionally, it is also possible that an underlying cardiomyopathy might have actually precipitated the spinal cord ischemia—although it is our hypothesis that the spinal stroke led to the development of NSM, we are limited to supposition and without definitive proof. It is likely that this phenomenon of myocardial stunning is underrecognized in patients who present with acute neurological disease and that the identification of this co-occurrence by echocardiography may inform the approach to supportive care during the patient's recovery period. Awareness of this phenomenon may encourage vigilant observation for rare but serious sequelae of NSM. Although not seen in this patient, these sequelae may include arrhythmias, hypotension, and cardiogenic shock, as well as dyskinesia of the left ventricle and the attendant risk of thrombus formation.

## Figures and Tables

**Table 1:** Pertinent laboratory data. Values and normal reference range noted for each laboratory test. All reported data is from time of presentation to emergency department unless otherwise specified.

Laboratory test	Value	Normal reference range
White blood cell count	9.2 × 10^3^/*μ*L	3.8–10.8 × 10^3^/*μ*L
Blood urea nitrogen	21 mg/dL	7–25 mg/dL
Creatinine	1.32 mg/dL	0.5–1.2 mg/dL
Point of care troponin *T* = 0 h	2.04 ng/mL	0.00–0.05 ng/mL
Point of care troponin at *T* = 3 h	1.72 ng/mL	0.00–0.05 ng/mL
Troponin I at *T* = 9 h	0.47 ng/mL	0.00–0.05 ng/mL
Point of care CKMB at *T* = 0 h	16.4 ng/mL	0.00–3.5 ng/mL
Point of care CKMB at *T* = 3 h	15.8 ng/mL	0.00–3.5 ng/mL
CKMB at *T* = 9 h	4.6 ng/mL	0.00–2.4 ng/mL
Lactate	1.9 mmol/L	0.5–2.2 mmol/L
International normalized ratio	1.1	0.9–1.1
Prothrombin time	14.1 seconds	11.6–14
Total cholesterol	255 mg/dL	0–200 mg/dL
High-density lipoprotein	48 mg/dL	30–60 mg/dL
Low-density lipoprotein	176 mg/dL	0–160 mg/dL
Triglycerides	154 mg/dL	10–150 mg/dL
